# Determinants of Career Satisfaction among Nursing Students

**DOI:** 10.1192/j.eurpsy.2026.11662

**Published:** 2026-07-31

**Authors:** A. Hrairi, N. Rmadi, M. Hajjaji, N. Kotti, N. E. H. Hachicha, N. Zaalani, A. Kamel, K. Jmal Hammami

**Affiliations:** 1 Occupational medicine department, Hedi Chaker university hospital; 2 Higher Institute of Nursing Sciences of Sfax, Sfax, Tunisia

## Abstract

**Introduction:**

Career satisfaction among nursing students is a key determinant of their motivation, professional commitment, and future retention in the nursing workforce. Identifying factors that influence career satisfaction is essential for improving educational strategies and ensuring a sustainable healthcare system.

**Objectives:**

This study aimed to assess the level of career satisfaction among nursing students and to identify the factors associated with it.

**Methods:**

A cross-sectional study was conducted among nursing students from two Institute of Nursing in Sfax, Tunisia. Data were collected online using a structured questionnaire including sociodemographic variables, academic characteristics. Satisfaction with nursing career was rated on a 10-point scale: A scale >5 was considered as high.

**Results:**

A total of 373 nursing students participated, 72.1% of whom were female, with a mean age of 21.07 ± 1.43 years. A total of 191 (51.2%) students have been certified in first aid. Furthermore, 88 (23.6%) students have been members of the Red Cross, and 70 (18.8%) have been Scout members. The majority of students 325 (87.1%) had willingly chosen nursing as a career. The mean score with nursing career was 7.18±1.86. A percentage of 84.7% had expressed a high satisfaction toward nursing. Career satisfaction was significantly associated with female gender (p=0.023 ; IC 95% [1.072-2.709]), first aid trainaing (p=0.005 ; IC 95%[1.185-2.698]) and the voluntary choice of nursing career (p=0.003 ; IC 95% [1.366-5.243]). It wasn’t associated with age, academic year, scout and red cross membership and living conditions.

**Conclusions:**

Career satisfaction among nursing students is influenced by both personal and academic factors. Strengthening academic support, fostering extracurricular involvement, and implementing targeted mentoring programs may help enhance satisfaction and promote professional commitment.

**Disclosure of Interest:**

None Declared

